# Extract from a Lecture on the Rights and Liabilities of the Physician and Surgeon

**Published:** 1855-03

**Authors:** Joel Parker

**Affiliations:** Professer of Medical Jurisprudence in Dartmouth College


Extract from a Lecture on the rights and liabilities of the Physician and Surgeon.—A physician is liable, also, to a civil action, for not exercising sufficient skill and care in the performance of his duty to his patients.

This is a principle fraught with danger to all medical men, and one requiring their profound consideration,—not that they are subjected to a greater responsibility than that imposed upon other professions and occupations, nor that they are more likely to be heedless in the performance of their duties, but because of the peculiar means which the patient has above all other litigants, of misrepresenting his case, and of moving the sympathies of the jurors who pass upon it.

The rules which regulate this liability are deduced from the general principle of the law, that where any person undertakes for a reward to perform a work for another, which requires skill for its accomplishment, he holds himself out as possessing the requisite knowledge and skill, and engages that he will exert and apply that skill in the performance of the work which he undertakes. And if he does not possess the skill which is requisite, and the employer, by reason of the want of it, suffers an injury; or, if possessing it, he does not use it, either from wilfulness or negligence, and damage is thereby sustained by the employer, the party who undertakes the performance of the service is liable to a suit for the recovery of the damages sustained by his incapacity, wilfulness or negligence. Thus the blacksmith who undertakes to shoe a horse, and ignorantly or negligently lames him, and the mechanic who, undertaking to repair a piece of machinery, fails from incapacity or carelessness to perform what he agrees to do, becomes answerable for the injury occasioned by his breach of duty. And so the attorney at law who, from want of a competent knowledge of his profession, or from neglect in applying his professional skill to the case committed to him, is liable at the suit of the client for the damage thereby sustained.

In like manner, and upon the same principle, a person who holds himself out as a physician, and assumes the care of a patient undertakes for the necessary skill to treat the disease in a proper manner, and to prescribe the medicines suited to counteract and cure, if perchance it may be cured, or to alleviate the pain of it, so far as that may reasonably be done, and if he fail in the performance of the duty thus devolved upon him by his employment, whether the failure be the result of ignorance of his profession, or want of due care in his prescriptions, if injury to the system of the patient, delay in the cure, or unnecessary pain can be shown to have resulted from his want of skill or care, the patient may have an action to recover his damages. The rule applies in full force in surgical practice, and the great danger of the medical man lies here. Actions are rare except in cases of surgery, and in cases of that character the plaintiff generally comes into court with some visible deformity, which, however, may have been occasioned, whether by his negligence, restlessness or wilfulness, excites sympathy, and being attributed to the mismanagement of the defendant, he is in great danger, even when he has been in no fault.

Several cases of this character have fallen under my own observation.

The inquiry naturally arises: How may the medical man best avoid the danger of loss—loss of money, and perhaps, to some extent, loss of reputation, through such prosecutions.

It has been suggested that, before acting in any case, he should require a bond or writing, exonerating him from all responsibility, or, perhaps, containing a direct stipulation not to prosecute a case of failure to effect a cure. Possibly such a document may have a tendency to restrain the party who has executed it from instituting a suit. But it admits of very grave doubt whether it could have any legal operation to exempt the physician from any responsibility.

Theoretically, he is liable only for want of skill or negligence. Practically, he is in danger from an unreasonable claim, and from the sympathy of the jury. The document, to be of any avail as a legal bar to a right of action, would be in effect a contract that the physician should not be liable even if he lacked skill, or was guilty of negligence. But it has been held that it is against the policy of the law to permit a party to contract for exemption from the legal consequences of his own fraud; and gross negligence has been held equivalent to fraud.

Whether the principle would be extended farther, and held to embrace such a contract with a physician, remains to be seen.

Such a precaution may do no harm, but from the nature of the case, it must be impracticable, in some instances, to procure such writing from the patient himself.

Another precaution, in cases where danger of a suit may be apprehended, is to secure sufficient evidence of the state and progress of the case from time to time, by an examination by other physicians, and by intelligent men not of the profession, and therefore not liable to a suspicion that their testimony is influenced by an *esprit de cörps.*

Another, when the patient is refractory or unreasonable, is to decline farther attendance, assigning the reason and giving reasonable time to procure another medical attendant. But whenever this course is resorted to there should be plenary evidence respecting the state in which the case was left.

Other measures will suggest themselves as adapted more or less to each case as it arises. Perhaps none of them can give a perfect assurance of exemption from unreasonable prosecutions.

But something may yet be hoped from an intelligent public opinion, which shall rebuke this sponging of the profession to indemnify the patient, in many instances, for the consequences of his own folly or negligence.—*Joel Parker, LL. D., Professer of Medical Jurisprudence in Dartmouth College.*

